# Crystal structures of a copper(II) and the isotypic nickel(II) and palladium(II) complexes of the ligand (*E*)-1-[(2,4,6-tri­bromo­phen­yl)diazen­yl]naphthalen-2-ol

**DOI:** 10.1107/S205698901601080X

**Published:** 2016-07-12

**Authors:** Souheyla Chetioui, Djamil-Azzeddine Rouag, Jean-Pierre Djukic, Christian G. Bochet, Rachid Touzani, Corinne Bailly, Aurélien Crochet, Katharina M. Fromm

**Affiliations:** a Unité de Recherche de Chimie de l’Environnement et Moléculaire Structurale (CHEMS), Faculté des Sciences Exactes, Département de Chimie, Université des Frères Mentouri Constantine, Constantine 25000, Algeria; b Laboratoire de Chimie et Systémique Organométallique (LCSOM), Institut de Chimie, Université de Strasbourg, UMR 7177., 4 rue Blaise Pascal, F-67070 Strasbourg Cedex, France; c Chemistry Department, University of Fribourg, Chemin du Musee 9, CH-1700 Fribourg, Switzerland; d Laboratoire de Chimie Appliquée et Environnement, LCAE-URAC18, COSTE, Faculté des Sciences, Université Mohamed Premier, BP524, 60000 Oujda, Morocco; e Faculté Pluridisciplinaire Nador BP 300, Selouane 62702, Nador, Morocco; f Service de Radiocristallographie, Institut de Chimie, Université de Strasbourg, UMR 7177, 67008 Strasbourg Cedex, France; g Fribourg Center for Nanomaterials, FriMat, University of Fribourg, Chemin du Musee 9, CH-1700 Fribourg, Switzerland

**Keywords:** crystal structures, copper(II), nickel(II), palladium(II), isotypic complexes, Cu⋯Br short contact, C—H⋯Br hydrogen bonds, C—H⋯π inter­actions

## Abstract

In the copper(II) complex, bis­{(*E*)-1-[(2,4,6-tri­bromo­phen­yl)diazen­yl]naph­thalen-2-olato}copper(II), [Cu(C_16_H_8_Br_3_N_2_O)_2_], (I), the metal cation is coord­inated by two N atoms and two O atoms from two bidentate (*E*)-1-[(2,4,6-tri­bromo­phen­yl)diazen­yl]naphthalen-2-olate ligands, forming a slightly distorted square-planar environment. In one of the ligands, the tri­bromo­benzene ring is inclined to the naphthalene ring system by 37.4 (5)°, creating a weak intra­molecular Cu⋯Br inter­action [3.134 (2) Å], while in the other ligand, the tri­bromo­benzene ring is inclined to the naphthalene ring system by 72.1 (6)°. In the isotypic nickel(II) and palladium(II) complexes, namely bis­{(*E*)-1-[(2,4,6-tri­bromo­phen­yl)diazen­yl]naphthalen-2-olato}nickel(II), [Ni(C_16_H_8_Br_3_N_2_O)_2_], (II), and bis­{(*E*)-1-[(2,4,6-tri­bromo­phen­yl)diazen­yl]naphthalen-2-olato}palladium(II), [Pd(C_16_H_8_Br_3_N_2_O)_2_], (III), respectively, the metal atoms are located on centres of inversion, hence the metal coordination spheres have perfect square-planar geometries. The tri­bromo­benzene rings are inclined to the naphthalene ring systems by 80.79 (18)° in (II) and by 80.8 (3)° in (III). In the crystal of (I), mol­ecules are linked by C—H⋯Br hydrogen bonds, forming chains along [010]. The chains are linked by C—H⋯π inter­actions, forming sheets parallel to (011). In the crystals of (II) and (III), mol­ecules are linked by C—H⋯π inter­actions, forming slabs parallel to (10-1). For the copper(II) complex (I), a region of disordered electron density was corrected for using the SQUEEZE routine in *PLATON* [Spek (2015 ▸). *Acta Cryst.* C**71**, 9–18]. The formula mass and unit-cell characteristics of the disordered solvent mol­ecules were not taken into account during refinement.

## Abstract 2

In the title copper(II) complex, the metal atom is coordinated by two N atoms and two O atoms from two bidentate (*E*)-1-[(2,4,6-tri­bromo­phen­yl)diazen­yl]naphthalen-2-ol ligands, forming a slightly distorted square-planar environment. In the isotypic nickel(II) and palladium(II) complexes, the metal atoms are located on centres of inversion, hence the metal coordination spheres have perfect square-planar geometries.

## Chemical context   

Recently, 1-phenyl­azo-2-naphthol derivatives have attracted attention because the phenyl­azo-naphtho­late group can provide *N*,*O*-bidentate chelation to stabilize transition or main group metal complexes. Azo-metal chelates have also attracted increasing attention due to their inter­esting electronic and geometrical features in connection with their applications in mol­ecular memory storage, non-linear optical elements and printing systems. Another advantage of complexes involving azo DNO’s (dyes and pigments) and transition metal ions is the possibility to obtain new compounds with biological activity (Thomas *et al.*, 2004 ▸; Reed *et al.*, 2006 ▸). Transition metals have also been used in the treatment of several diseases, as metal complexes which are capable of cleaving DNA under physiological conditions are of inter­est in the development of metal-based anti­cancer agents. This is an impetus for chemists to develop innovative strat­egies for the preparation of more effective, target-specific and preferably non-covalently bound anti­cancer drugs (Chen *et al.*, 2010 ▸; Cvek *et al.*, 2008 ▸).
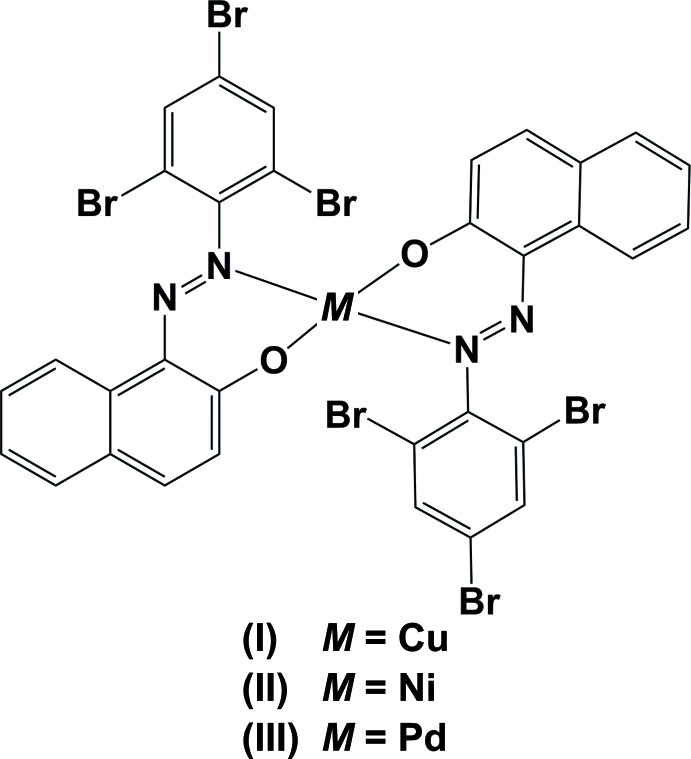


Being inter­ested in the synthesis and preparation of metal complexes bearing such ligands, we have successfully synthesized and structurally characterized Cu^II^ complexes with *N*,*O*-bidentate phenyl­azo-naphtho­late ligands (Chetioui *et al.*, 2015*a* ▸,*b* ▸). In this work we are involved in the colour-generation mechanism of azo pigments typically characterized by the chromophore of the azo group (–N=N–) (Chetioui *et al.*, 2013*c* ▸,*d* ▸) in order to synthesize new complexes with Cu(OAc)_2_·H_2_O, Ni(OAc)_2_·H_2_O, and Pd(OAc)_2_·H_2_O. We report herein on the synthesis and crystal structures of the title complexes, (I)–(III), of the ligand (*E*)-1-[(2,4,6-tri­bromo­phen­yl)diazen­yl]naphthalen-2-ol, whose crystal structure has been described previously (Chetioui *et al.*, 2013*c* ▸).

## Structural commentary   

**Figure 1:**
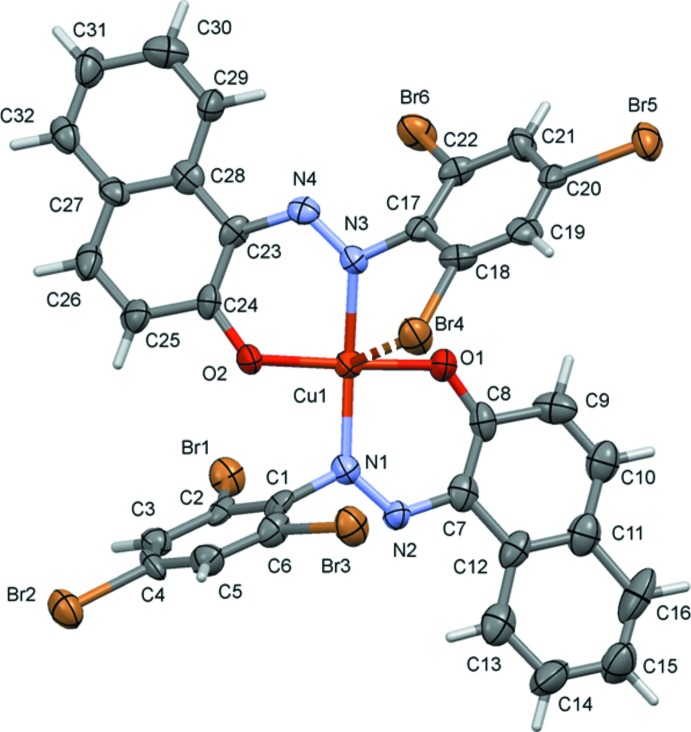
The mol­ecular structure of compound (I), with atom labelling and 50% probability displacement ellipsoids. The intra­molecular Cu⋯Br contact is shown as a dashed line (details are given in Table 1 ▸).

In all three compounds the ligand (*E*)-1-[(2,4,6-tri­bromo­phen­yl)diazen­yl]naphthalen-2-ol (Chetioui *et al.*, 2013*c* ▸) coordinates in a *N*,*O*-bidentate manner. The metal atoms are coordinated by two oxygen atoms in a *trans* position of the C—O^−^ function and two nitro­gen atoms in a *trans* position of the N=N function. In compound (I), Fig. 1 ▸, the values of the angles involving the copper and the two oxygen and two nitro­gen atoms (Table 1 ▸) indicate that the geometry of the coordination polyhedron is distorted square-planar. It has a τ_4_ value of 0.15 [Yang *et al.*, 2007 ▸; extreme configurations: 0.00 for square-pyramidal (SQP) and 1.00 for tetrahedral (TET); 0.85 for trigonal–pyramidal (TRP)]. In one of the ligands, the tri­bromo­benzene ring (C17–C22) is inclined to the naphthalene ring system (C23–C32) by 37.4 (5)°, creating a weak intra­molecular Cu⋯Br inter­action [Cu1⋯Br4 = 3.134 (2) Å]. In the other ligand, the tri­bromo­benzene ring (C1–C6) is almost normal to the naphthalene ring system (C7-C16), making a dihedral angle of 72.1 (6)°. A similar short intra­molecular metal–halogen contact has been observed in the centrosymmetric complex bis­(1-[(*E*)-(2-chloro­phen­yl)diazen­yl]naphthalen-2-olato)copper(II), *viz.* Cu⋯Cl = 3.153 (1) Å (Benaouida *et al.*, 2013 ▸), and the chloro­benzene ring is inclined to the naphthalene ring system by 32.72 (12)°.

**Figure 2:**
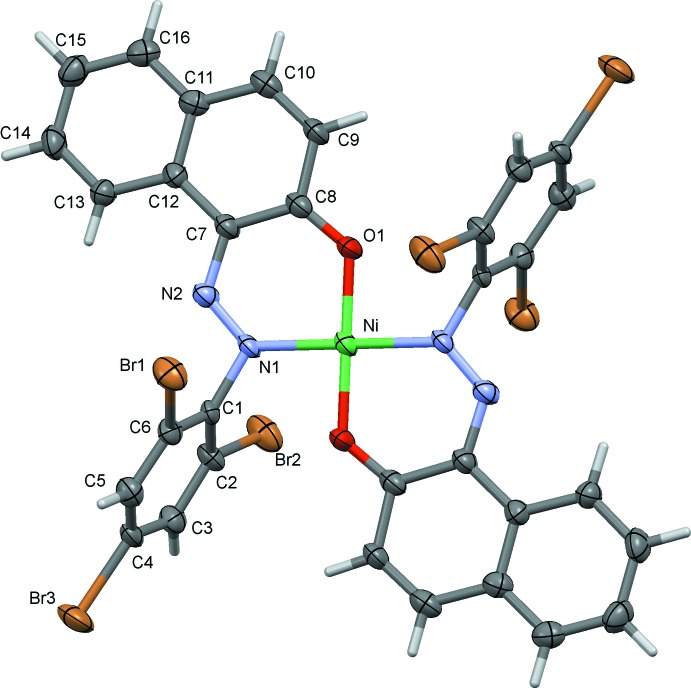
The mol­ecular structure of compound (II), with atom labelling and 50% probability displacement ellipsoids. The unlabelled atoms are related to the labelled atoms by the symmetry operation (−*x* + 1, −*y* + 1, −*z* + 1).

**Figure 3:**
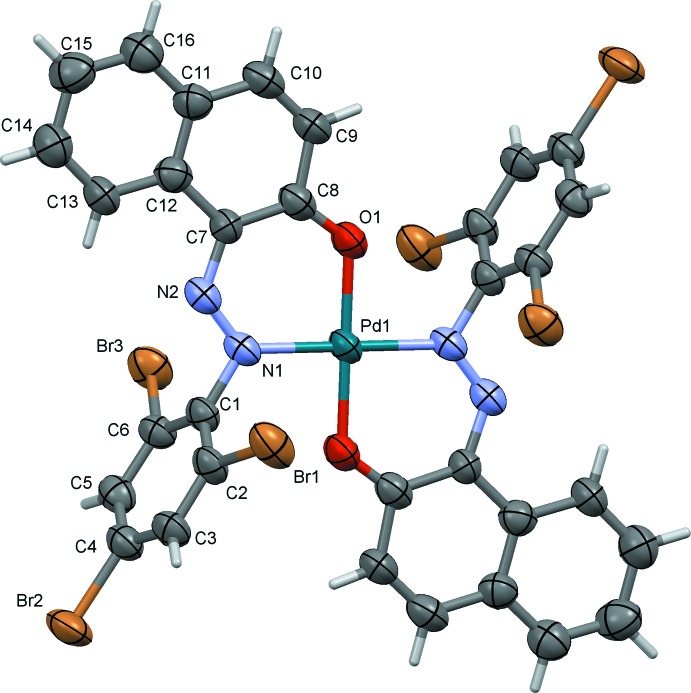
The mol­ecular structure of compound (III), with atom labelling and 50% probability displacement ellipsoids. The unlabelled atoms are related to the labelled atoms by the symmetry operation (−*x*, −*y*, −*z*).

Compounds (II) and (III), the nickel(II) (Fig. 2 ▸, Table 2 ▸) and palladium(II) (Fig. 3 ▸, Table 3 ▸) complexes, respectively, are isotypic. The metal atoms are each located on inversion centres, coordinating in a bidentate fashion to the N and O atoms of the ligand, hence the metal coordination spheres have perfect square-planar geometry. The tri­bromo­benzene rings (C1–C6) are almost normal to the naphthalene ring systems (C7–C16) with a dihedral angle of 80.79 (18)° in (II) and 80.8 (3)° in (III).

## Supra­molecular features   

**Figure 4:**
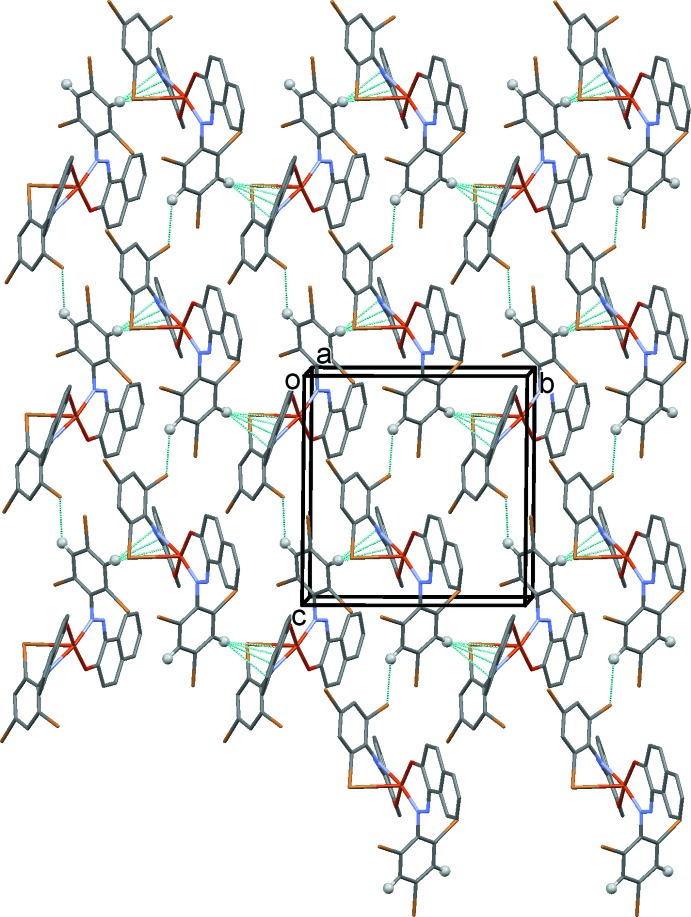
The crystal packing of compound (I), viewed along the *a* axis. The inter­molecular inter­actions are shown as dashed lines (see Table 4 ▸ for details), and for clarity only the H atoms involved in these inter­actions have been included.

As shown in Fig. 4 ▸, in the crystal of compound (I), mol­ecules are linked by C—H⋯Br hydrogen bonds, forming chains along [001]. The chains are linked by C—H⋯π inter­actions, forming sheets lying parallel to (011). Details of these inter­actions are given in Table 4 ▸.

**Figure 5:**
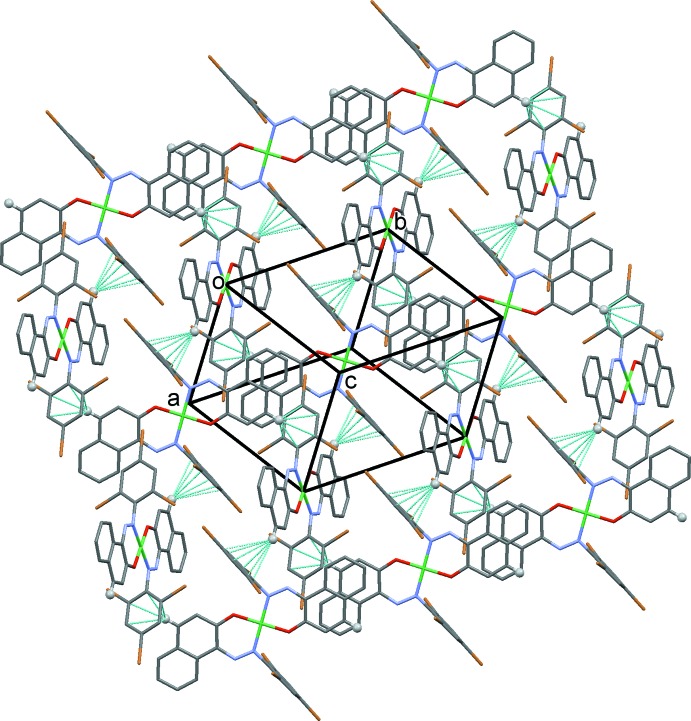
The crystal packing of compound (II), viewed along the normal to (10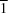). The inter­molecular inter­actions are shown as dashed lines (see Table 5 ▸ for details), and for clarity only the H atoms involved in these inter­actions have been included.

The crystal packing in compound (II) [and isotypic compound (III)] is illustrated in Fig. 5 ▸. Mol­ecules are linked by C—H⋯π inter­actions, forming slabs lying parallel to (10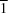). Details of the inter­molecular inter­actions are given in Table 5 ▸ for (II) and Table 6 ▸ for (III).

## Database survey   

**Figure 6:**
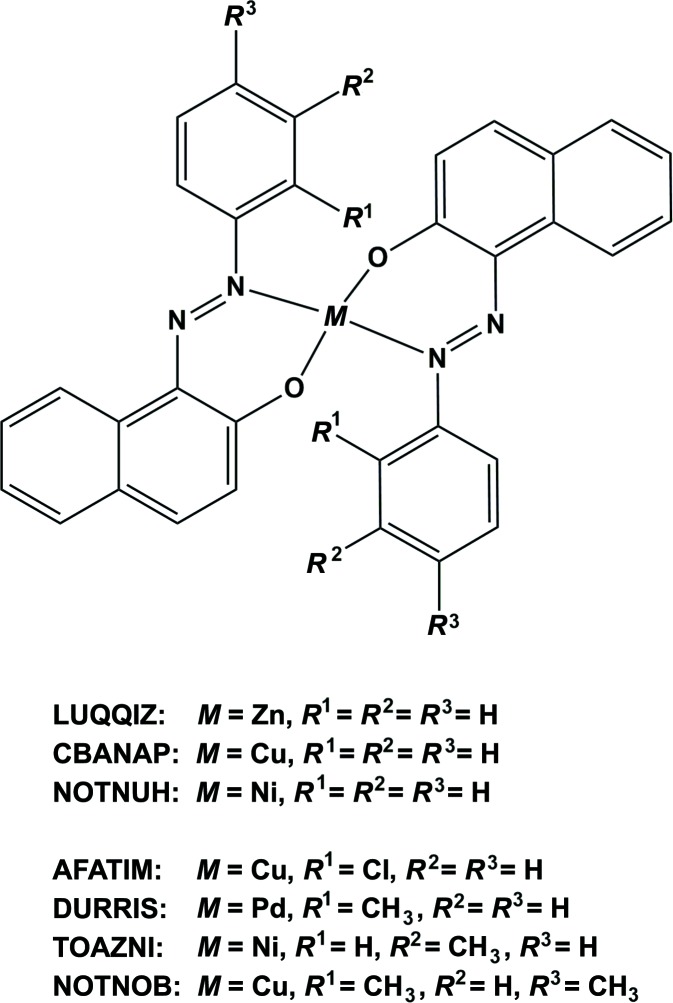
The results of the database search (CSD; Groom *et al.*, 2016 ▸) for four-coordinate metal complexes of the ligand (*E*)-1-(phenyl­diazen­yl)naphthalen-2-ol and its derivatives.

In the title ligand (*E*)-1-[(2,4,6-tri­bromo­phen­yl)diazen­yl]naphthalen-2-ol (CSD refcode AFOFIM; Chetioui *et al.*, 2013*c* ▸) the benzene ring is inclined to the naphthalene ring system by 33.80 (16)°. A search of the Cambridge Structural Database (Version 5.37, update February 2016; Groom *et al.*, 2016 ▸) for square-planar metal complexes of (*E*)-1-(phenyl­diazen­yl)naphthalen-2-ol and its derivatives gave seven hits (Fig. 6 ▸). They include a zinc(II) complex of the ligand (*E*)-1-(phenyl­diazen­yl)naphthalen-2-ol (LUQQIZ; Gallegos *et al.*, 2015 ▸), where the zinc atom has a distorted trigonal–pyramidal configuration with a τ_4_ parameter of 0.77. In the two ligands, the phenyl rings are inclined to the naphthalene ring systems by 11.4 (2) and 9.2 (3)°. Among the other six complexes, in which the metal atoms are all located on inversion centres, there are three copper(II) complexes with the ligands (*E*)-1-(phenyl­diazen­yl)naphthalen-2-ol (refcode CBANAP; Jarvis, 1961 ▸), (*E*)-1-(2-chloro­phen­yl)diazen­yl]naphthalen-2-ol (AFATIM; Benaouida *et al.*, 2013 ▸) and (*E*)-1-(2,4-di­methyl­phen­yl)diazen­yl]naphthalen-2-ol (NOTNOB; Ferreira *et al.*, 2015 ▸); two nickel complexes with the ligands (*E*)-1-(phenyl­diazen­yl)naphthalen-2-ol (NOTNUH; Ferreira *et al.*, 2015 ▸) and (*E*)-1-(3-methyl­phen­yl)diazen­yl]naphthalen-2-ol (TOAZNI; Alcock *et al.*, 1968 ▸); and one palladium complex with the ligand [(*E*)-1-(2-methyl­phen­yl)diazen­yl]naphthalen-2-ol (DURRIS; Lin *et al.*, 2010 ▸). The orientation of the phen­yl/benzene ring with respect to the naphthalene ring system varies quite considerably. In the palladium complex (DURRIS) and the copper complex (NOTNOB), where the benzene ring has a methyl group in the *ortho* position, the benzene ring is inclined to the naphthalene ring system by 74.41 (4) and 83.87 (6)°, respectively. In the other four complexes, the corresponding dihedral angles are 19.12 and 32.72 (12)° for the copper complexes CBANAP and AFATIM, respectively, and 24.06 (15) and *ca* 35.56° for the nickel complexes NOTNUH and TOAZNI, respectively.

## Synthesis and crystallization   

The title compounds were synthesized by the following procedure: (*E*)-1-[(2,4,6-tri­bromo­phen­yl)diazen­yl]-naphthal­en-2-ol (2.0 mmol) and *M*(OAc)_2_·H_2_O (1.0 mmol; where *M* = Cu, Ni, Pd) was stirred at 298 K in a mixture of THF/MeOH (10/10 ml) for 24 h. The solvents were removed under vacuum and the residue was washed twice with hexane to give dark solids. The resulting solids were crystallized from CH_2_Cl_2_ to yield red block-like crystals for (I), black prismatic crystals for (II) and dark-red plate-like crystals for (III).

## Refinement details   

Crystal data, data collection and structure refinement details are summarized in Table 7 ▸. For all three compounds the C-bound H atoms were included in calculated positions and refined as riding: C—H = 0.95 Å for (I) and (II) and 0.93 Å for (III), with *U*_iso_(H) = 1.2*U*_eq_(C). For the copper(II) complex (I), a region of disordered electron density was corrected for using the SQUEEZE routine in *PLATON* (Spek, 2015 ▸). The formula mass and unit-cell characteristics of the disordered solvent mol­ecules were not taken into account during refinement. This complex crystallizes in the monoclinic space group *P*2_1_, with the Flack parameter = −0.006 (14).

## Supplementary Material

Crystal structure: contains datablock(s) global, I, II, III. DOI: 10.1107/S205698901601080X/su5299sup1.cif

Structure factors: contains datablock(s) I. DOI: 10.1107/S205698901601080X/su5299Isup3.hkl

Structure factors: contains datablock(s) II. DOI: 10.1107/S205698901601080X/su5299IIsup4.hkl

Structure factors: contains datablock(s) III. DOI: 10.1107/S205698901601080X/su5299IIIsup2.hkl

CCDC references: 1490056, 1490055, 1490054

Additional supporting information:  crystallographic information; 3D view; checkCIF report

## Figures and Tables

**Table 1:** Selected geometric parameters (Å, °) for (I)

Cu1—Br4	3.134 (2)	Cu1—O1	1.892 (9)
Cu1—N1	1.947 (12)	Cu1—O2	1.888 (8)
Cu1—N3	1.970 (11)		
			
O2—Cu1—O1	169.4 (4)	O2—Cu1—N3	87.6 (4)
O2—Cu1—N1	91.3 (4)	O1—Cu1—N3	92.1 (4)
O1—Cu1—N1	90.9 (4)	N1—Cu1—N3	169.3 (5)

**Table 2:** Selected geometric parameters (Å, °) for (II)

Ni—N1	1.876 (3)	Ni—O1	1.821 (3)
			
O1—Ni—O1^i^	180	O1^i^—Ni—N1	87.41 (14)
O1—Ni—N1	92.59 (14)	N1—Ni—N1^i^	180

Symmetry code: (i) 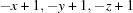.

**Table 3:** Selected geometric parameters (Å, °) for (III)

Pd1—N1	2.004 (5)	Pd1—O1	1.972 (5)
			
O1—Pd1—O1^i^	180	O1^i^—Pd1—N1	88.7 (2)
O1—Pd1—N1	91.3 (2)	N1—Pd1—N1^i^	180

Symmetry code: (i) 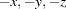.

**Table 4:** Hydrogen-bond geometry (Å, °) for (I) *Cg*1 is the centroid of the C27–C32 ring.

*D*—H⋯*A*	*D*—H	H⋯*A*	*D*⋯*A*	*D*—H⋯*A*
C5—H5⋯Br6^i^	0.95	2.75	3.546 (15)	142
C3—H3⋯*Cg*1^ii^	0.95	2.99	3.729 (15)	136

Symmetry codes: (i) 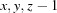; (ii) 
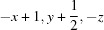
.

**Table 5:** Hydrogen-bond geometry (Å, °) for (II) *Cg*2 is the centroid of the C1–C6 ring.

*D*—H⋯*A*	*D*—H	H⋯*A*	*D*⋯*A*	*D*—H⋯*A*
C10—H10⋯*Cg*2^ii^	0.95	2.71	3.391 (5)	130

Symmetry code: (ii) 
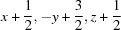
.

**Table 6:** Hydrogen-bond geometry (Å, °) for (III) *Cg*2 is the centroid of the C1–C6 ring.

*D*—H⋯*A*	*D*—H	H⋯*A*	*D*⋯*A*	*D*—H⋯*A*
C10—H10⋯*Cg*2^ii^	0.93	2.70	3.371 (8)	129

Symmetry code: (ii) 
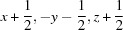
.

**Table 7:** Experimental details

	(I)	(II)	(III)
Crystal data			
Chemical formula	[Cu(C_16_H_8_Br_3_N_2_O)_2_]	[Ni(C_16_H_8_Br_3_N_2_O)_2_]	[Pd(C_16_H_8_Br_3_N_2_O)_2_]
*M*_r_	1031.49	1026.66	1074.35
Crystal system, space group	Monoclinic, *P*2_1_	Monoclinic, *P*2_1_/*n*	Monoclinic, *P*2_1_/*n*
Temperature (K)	173	173	200
*a*, *b*, *c* (Å)	11.9423 (7), 12.1314 (10), 12.8974 (10)	11.0909 (6), 12.4571 (6), 12.5382 (7)	11.1896 (8), 12.4540 (8), 12.5511 (9)
β (°)	107.032 (4)	107.820 (2)	107.749 (5)
*V* (Å^3^)	1786.6 (2)	1649.17 (15)	1665.8 (2)
*Z*	2	2	2
Radiation type	Mo *K*α	Mo *K*α	Cu *K*α
μ (mm^−1^)	7.36	7.89	13.23
Crystal size (mm)	0.20 × 0.15 × 0.06	0.30 × 0.22 × 0.06	0.12 × 0.09 × 0.03

Data collection			
Diffractometer	Nonius KappaCCD	Nonius KappaCCD	STOE *IPDS* 2T
Absorption correction	Multi-scan (*MULABS*; Spek, 2009)	Multi-scan (*MULABS*; Spek, 2009)	Multi-scan (*MULABS*; Spek, 2009)
*T*_min_, *T*_max_	0.311, 0.386	0.151, 0.317	0.360, 1.000
No. of measured, independent and observed [*I* > 2σ(*I*)] reflections	14985, 7819, 4785	11360, 3745, 2214	13003, 2895, 2371
*R*_int_	0.077	0.094	0.142
(sin θ/λ)_max_ (Å^−1^)	0.650	0.649	0.600

Refinement			
*R*[*F* ^2^ > 2σ(*F* ^2^)], *wR*(*F* ^2^), *S*	0.064, 0.140, 0.96	0.043, 0.096, 0.95	0.057, 0.170, 1.11
No. of reflections	7819	3745	2895
No. of parameters	388	205	206
No. of restraints	2	0	0
H-atom treatment	H-atom parameters constrained	H-atom parameters constrained	H-atom parameters constrained
Δρ_max_, Δρ_min_ (e Å^−3^)	0.59, −0.58	0.57, −0.66	0.88, −1.10
Absolute structure	Flack *x* determined using 1648 quotients [(*I* ^+^)-(*I* ^-^)]/[(*I* ^+^)+(*I* ^-^)] (Parsons *et al.*, 2013 ▸)	–	–
Absolute structure parameter	−0.006 (14)	–	–

Computer programs: *COLLECT* (Nonius, 1998 ▸), *X-AREA* and *X-RED32* (Stoe & Cie, 2002 ▸), *DENZO* (Otwinowski & Minor, 1997 ▸), *SIR97* (Altomare *et al.*, 1999 ▸), *SHELXS2014* (Sheldrick, 2008 ▸), *SHELXL2014* (Sheldrick, 2015 ▸), *Mercury* (Macrae *et al.*, 2008 ▸) and *PLATON* (Spek, 2009 ▸).

## supplementary crystallographic information

### (I) Bis{(E)-1-[(2,4,6-tribromophenyl)diazenyl]naphthalen-2-olato}copper(II) Crystal data

**Table:**

[Cu(C_16_H_8_Br_3_N_2_O)_2_]	*F*(000) = 982
*M**_r_* = 1031.49	*D*_x_ = 1.917 Mg m^−^^3^
Monoclinic, *P*2_1_	Mo *K*α radiation, λ = 0.71073 Å
*a* = 11.9423 (7) Å	Cell parameters from 19031 reflections
*b* = 12.1314 (10) Å	θ = 1.0–27.5°
*c* = 12.8974 (10) Å	µ = 7.36 mm^−^^1^
β = 107.032 (4)°	*T* = 173 K
*V* = 1786.6 (2) Å^3^	Block, red
*Z* = 2	0.20 × 0.15 × 0.06 mm

### (I) Bis{(E)-1-[(2,4,6-tribromophenyl)diazenyl]naphthalen-2-olato}copper(II) Data collection

**Table:**

Nonius KappaCCD diffractometer	7819 independent reflections
Radiation source: sealed tube	4785 reflections with *I* > 2σ(*I*)
Graphite monochromator	*R*_int_ = 0.077
phi and ω scans	θ_max_ = 27.5°, θ_min_ = 2.4°
Absorption correction: multi-scan (MULABS; Spek, 2009)	*h* = −15→15
*T*_min_ = 0.311, *T*_max_ = 0.386	*k* = −15→15
14985 measured reflections	*l* = −16→16

### (I) Bis{(E)-1-[(2,4,6-tribromophenyl)diazenyl]naphthalen-2-olato}copper(II) Refinement

**Table:**

Refinement on *F*^2^	Secondary atom site location: difference Fourier map
Least-squares matrix: full	Hydrogen site location: inferred from neighbouring sites
*R*[*F*^2^ > 2σ(*F*^2^)] = 0.064	H-atom parameters constrained
*wR*(*F*^2^) = 0.140	*w* = 1/[σ^2^(*F*_o_^2^) + (0.0639*P*)^2^] where *P* = (*F*_o_^2^ + 2*F*_c_^2^)/3
*S* = 0.96	(Δ/σ)_max_ < 0.001
7819 reflections	Δρ_max_ = 0.59 e Å^−^^3^
388 parameters	Δρ_min_ = −0.58 e Å^−^^3^
2 restraints	Absolute structure: Flack *x* determined using 1648 quotients [(*I*^+^)-(*I*^-^)]/[(*I*^+^)+(*I*^-^)] (Parsons *et al.*, 2013)
Primary atom site location: structure-invariant direct methods	Absolute structure parameter: −0.006 (14)

### (I) Bis{(E)-1-[(2,4,6-tribromophenyl)diazenyl]naphthalen-2-olato}copper(II) Special details

**Table:**

Geometry. All esds (except the esd in the dihedral angle between two l.s. planes) are estimated using the full covariance matrix. The cell esds are taken into account individually in the estimation of esds in distances, angles and torsion angles; correlations between esds in cell parameters are only used when they are defined by crystal symmetry. An approximate (isotropic) treatment of cell esds is used for estimating esds involving l.s. planes.

### (I) Bis{(E)-1-[(2,4,6-tribromophenyl)diazenyl]naphthalen-2-olato}copper(II) Fractional atomic coordinates and isotropic or equivalent isotropic displacement parameters (Å^2^)

**Table:**

	*x*	*y*	*z*	*U*_iso_*/*U*_eq_	
C1	0.8599 (11)	1.0263 (11)	−0.0117 (12)	0.032 (3)	
C2	0.7637 (10)	1.0951 (10)	−0.0438 (11)	0.029 (3)	
C3	0.6867 (12)	1.0894 (11)	−0.1484 (12)	0.034 (3)	
H3	0.6209	1.1370	−0.1709	0.041*	
C4	0.7093 (12)	1.0134 (11)	−0.2171 (11)	0.036 (3)	
C5	0.8009 (12)	0.9443 (13)	−0.1921 (12)	0.041 (4)	
H5	0.8118	0.8927	−0.2438	0.049*	
C6	0.8812 (11)	0.9500 (11)	−0.0865 (13)	0.038 (4)	
C7	1.1218 (11)	1.0834 (10)	0.2004 (11)	0.037 (4)	
C8	1.1126 (12)	1.0508 (12)	0.3013 (11)	0.038 (4)	
C9	1.2090 (15)	1.0731 (12)	0.3969 (14)	0.050 (4)	
H9	1.2028	1.0532	0.4662	0.060*	
C10	1.3075 (13)	1.1215 (13)	0.3890 (14)	0.050 (3)	
H10	1.3702	1.1346	0.4529	0.060*	
C11	1.3195 (13)	1.1534 (13)	0.2865 (15)	0.050 (3)	
C12	1.2277 (12)	1.1344 (11)	0.1930 (14)	0.042 (4)	
C13	1.2435 (13)	1.1699 (12)	0.0916 (15)	0.051 (4)	
H13	1.1810	1.1617	0.0267	0.061*	
C14	1.3475 (13)	1.2156 (15)	0.0870 (17)	0.066 (5)	
H14	1.3548	1.2383	0.0188	0.079*	
C15	1.4404 (14)	1.2291 (16)	0.1775 (16)	0.060 (5)	
H15	1.5122	1.2576	0.1713	0.072*	
C16	1.4305 (13)	1.2026 (16)	0.274 (2)	0.075 (6)	
H16	1.4947	1.2150	0.3369	0.090*	
C17	0.9418 (11)	0.7896 (11)	0.3903 (12)	0.034 (3)	
C18	1.0263 (12)	0.7317 (12)	0.3582 (12)	0.039 (4)	
C19	1.1197 (13)	0.6785 (11)	0.4318 (13)	0.042 (4)	
H19	1.1765	0.6396	0.4077	0.050*	
C20	1.1273 (11)	0.6835 (12)	0.5348 (13)	0.039 (4)	
C21	1.0499 (11)	0.7419 (11)	0.5769 (12)	0.038 (3)	
H21	1.0600	0.7456	0.6526	0.046*	
C22	0.9586 (11)	0.7938 (11)	0.5032 (12)	0.033 (3)	
C23	0.6472 (5)	0.8679 (7)	0.2481 (6)	0.032 (3)	
C24	0.6455 (5)	0.9058 (8)	0.1459 (7)	0.033 (3)	
C25	0.5394 (7)	0.9308 (8)	0.0697 (6)	0.040 (4)	
H25	0.5383	0.9567	−0.0001	0.047*	
C26	0.4351 (5)	0.9179 (8)	0.0956 (6)	0.044 (4)	
H26	0.3627	0.9350	0.0435	0.053*	
C27	0.4369 (5)	0.8800 (8)	0.1978 (7)	0.035 (3)	
C28	0.5429 (7)	0.8550 (7)	0.2740 (6)	0.035 (3)	
C29	0.5396 (11)	0.8189 (12)	0.3776 (13)	0.042 (4)	
H29	0.6110	0.7992	0.4299	0.050*	
C30	0.4378 (14)	0.8113 (13)	0.4055 (14)	0.053 (4)	
H30	0.4391	0.7902	0.4767	0.063*	
C31	0.3284 (12)	0.8359 (12)	0.3248 (14)	0.048 (4)	
H31	0.2568	0.8300	0.3424	0.057*	
C32	0.3279 (12)	0.8675 (12)	0.2240 (12)	0.041 (4)	
H32	0.2557	0.8815	0.1703	0.049*	
N1	0.9383 (9)	1.0290 (9)	0.0989 (10)	0.035 (3)	
N2	1.0368 (9)	1.0745 (9)	0.1038 (9)	0.032 (3)	
N3	0.8497 (9)	0.8449 (9)	0.3100 (9)	0.031 (3)	
N4	0.7460 (9)	0.8232 (8)	0.3214 (9)	0.031 (3)	
O1	1.0249 (7)	0.9986 (8)	0.3181 (8)	0.040 (2)	
O2	0.7408 (7)	0.9216 (8)	0.1119 (7)	0.038 (2)	
Cu1	0.88988 (14)	0.94978 (13)	0.20990 (14)	0.0328 (4)	
Br1	0.73408 (13)	1.19596 (12)	0.05505 (13)	0.0456 (4)	
Br2	0.60688 (14)	1.00205 (14)	−0.36393 (13)	0.0535 (5)	
Br3	1.01318 (13)	0.85864 (13)	−0.05111 (13)	0.0473 (4)	
Br4	1.01312 (13)	0.72221 (13)	0.20691 (13)	0.0484 (4)	
Br5	1.25913 (13)	0.61791 (13)	0.64036 (14)	0.0482 (4)	
Br6	0.85805 (13)	0.88104 (13)	0.55982 (13)	0.0506 (4)	

### (I) Bis{(E)-1-[(2,4,6-tribromophenyl)diazenyl]naphthalen-2-olato}copper(II) Atomic displacement parameters (Å^2^)

**Table:**

	*U*^11^	*U*^22^	*U*^33^	*U*^12^	*U*^13^	*U*^23^
C1	0.024 (7)	0.031 (7)	0.042 (9)	−0.010 (6)	0.014 (6)	−0.003 (7)
C2	0.023 (6)	0.020 (7)	0.045 (9)	−0.008 (6)	0.012 (6)	−0.003 (6)
C3	0.037 (8)	0.033 (8)	0.032 (9)	0.002 (6)	0.010 (7)	0.005 (7)
C4	0.041 (8)	0.031 (8)	0.023 (8)	−0.005 (7)	−0.008 (6)	0.008 (7)
C5	0.043 (8)	0.046 (9)	0.035 (9)	−0.006 (7)	0.014 (7)	−0.007 (7)
C6	0.033 (7)	0.027 (7)	0.057 (11)	0.006 (6)	0.019 (7)	0.000 (7)
C7	0.033 (8)	0.027 (8)	0.050 (10)	0.009 (6)	0.009 (7)	−0.002 (7)
C8	0.032 (8)	0.038 (8)	0.038 (10)	0.008 (7)	0.000 (7)	−0.004 (7)
C9	0.073 (11)	0.040 (9)	0.042 (11)	−0.007 (8)	0.025 (9)	−0.002 (8)
C10	0.040 (6)	0.048 (7)	0.060 (8)	0.002 (5)	0.012 (6)	−0.010 (6)
C11	0.040 (6)	0.048 (7)	0.060 (8)	0.002 (5)	0.012 (6)	−0.010 (6)
C12	0.032 (8)	0.034 (9)	0.061 (12)	−0.005 (7)	0.014 (8)	−0.007 (8)
C13	0.042 (9)	0.037 (9)	0.071 (14)	0.007 (7)	0.014 (9)	−0.001 (8)
C14	0.044 (10)	0.072 (13)	0.088 (15)	−0.007 (9)	0.028 (10)	0.018 (12)
C15	0.039 (9)	0.080 (13)	0.064 (13)	−0.003 (9)	0.020 (9)	−0.009 (11)
C16	0.035 (9)	0.066 (12)	0.12 (2)	−0.018 (9)	0.020 (10)	−0.031 (13)
C17	0.032 (7)	0.025 (7)	0.046 (10)	0.002 (6)	0.014 (7)	0.013 (7)
C18	0.047 (8)	0.036 (8)	0.039 (9)	−0.004 (7)	0.022 (7)	0.005 (7)
C19	0.045 (9)	0.031 (8)	0.053 (11)	0.006 (7)	0.019 (8)	0.019 (8)
C20	0.027 (7)	0.042 (9)	0.049 (10)	0.000 (7)	0.012 (7)	0.024 (8)
C21	0.033 (7)	0.039 (8)	0.037 (9)	0.010 (7)	0.003 (7)	0.008 (7)
C22	0.039 (8)	0.025 (7)	0.043 (9)	0.003 (6)	0.024 (7)	−0.004 (7)
C23	0.028 (7)	0.028 (7)	0.045 (9)	0.006 (6)	0.018 (6)	−0.007 (7)
C24	0.025 (7)	0.034 (8)	0.034 (9)	−0.004 (6)	0.001 (6)	−0.007 (6)
C25	0.034 (8)	0.043 (9)	0.038 (9)	−0.013 (7)	0.006 (7)	−0.007 (7)
C26	0.029 (8)	0.036 (8)	0.059 (11)	−0.007 (7)	−0.001 (7)	0.004 (7)
C27	0.040 (8)	0.029 (7)	0.034 (8)	0.002 (7)	0.007 (6)	0.009 (7)
C28	0.044 (8)	0.024 (7)	0.034 (9)	−0.002 (6)	0.009 (7)	−0.008 (6)
C29	0.025 (7)	0.048 (9)	0.050 (10)	0.002 (7)	0.008 (6)	0.011 (7)
C30	0.072 (11)	0.045 (9)	0.050 (11)	0.004 (9)	0.031 (9)	0.006 (8)
C31	0.029 (8)	0.046 (10)	0.064 (12)	0.009 (7)	0.006 (7)	0.007 (8)
C32	0.043 (8)	0.033 (8)	0.037 (9)	−0.004 (7)	−0.003 (7)	−0.001 (7)
N1	0.032 (6)	0.038 (7)	0.032 (7)	0.000 (5)	0.005 (5)	0.005 (5)
N2	0.027 (6)	0.043 (7)	0.025 (7)	−0.008 (5)	0.006 (5)	−0.003 (5)
N3	0.029 (6)	0.034 (6)	0.030 (7)	0.001 (5)	0.005 (5)	0.006 (5)
N4	0.038 (7)	0.022 (6)	0.032 (7)	−0.002 (5)	0.011 (5)	0.000 (5)
O1	0.030 (5)	0.052 (6)	0.035 (6)	−0.007 (5)	0.004 (4)	0.003 (5)
O2	0.031 (5)	0.048 (6)	0.027 (6)	−0.009 (4)	−0.002 (4)	0.007 (4)
Cu1	0.0300 (8)	0.0336 (9)	0.0334 (10)	−0.0028 (7)	0.0071 (7)	0.0030 (8)
Br1	0.0499 (9)	0.0385 (8)	0.0471 (10)	0.0081 (7)	0.0122 (7)	−0.0023 (7)
Br2	0.0559 (9)	0.0580 (10)	0.0374 (10)	−0.0012 (9)	−0.0006 (7)	0.0009 (8)
Br3	0.0452 (8)	0.0488 (9)	0.0470 (10)	0.0147 (8)	0.0118 (7)	−0.0006 (8)
Br4	0.0449 (8)	0.0618 (11)	0.0390 (10)	0.0137 (8)	0.0131 (7)	−0.0007 (8)
Br5	0.0397 (8)	0.0465 (9)	0.0494 (11)	0.0064 (7)	−0.0013 (7)	0.0111 (8)
Br6	0.0586 (10)	0.0565 (10)	0.0397 (10)	0.0181 (8)	0.0188 (8)	0.0027 (8)

### (I) Bis{(E)-1-[(2,4,6-tribromophenyl)diazenyl]naphthalen-2-olato}copper(II) Geometric parameters (Å, º)

**Table:**

C1—C2	1.382 (17)	C18—Br4	1.914 (15)
C1—C6	1.413 (19)	C19—C20	1.31 (2)
C1—N1	1.460 (18)	C19—H19	0.9500
C2—C3	1.395 (19)	C20—C21	1.39 (2)
C2—Br1	1.874 (13)	C20—Br5	1.926 (13)
C3—C4	1.36 (2)	C21—C22	1.372 (18)
C3—H3	0.9500	C21—H21	0.9500
C4—C5	1.341 (19)	C22—Br6	1.900 (13)
C4—Br2	1.935 (13)	C23—N4	1.388 (12)
C5—C6	1.42 (2)	C23—C24	1.3900
C5—H5	0.9500	C23—C28	1.3900
C6—Br3	1.870 (13)	C24—O2	1.348 (10)
C7—N2	1.361 (16)	C24—C25	1.3900
C7—C8	1.394 (12)	C25—C26	1.3900
C7—C12	1.436 (19)	C25—H25	0.9500
C8—O1	1.296 (16)	C26—C27	1.3900
C8—C9	1.44 (2)	C26—H26	0.9500
C9—C10	1.34 (2)	C27—C28	1.3900
C9—H9	0.9500	C27—C32	1.445 (16)
C10—C11	1.42 (2)	C28—C29	1.418 (16)
C10—H10	0.9500	C29—C30	1.37 (2)
C11—C12	1.39 (2)	C29—H29	0.9500
C11—C16	1.50 (2)	C30—C31	1.44 (2)
C12—C13	1.44 (2)	C30—H30	0.9500
C13—C14	1.38 (2)	C31—C32	1.35 (2)
C13—H13	0.9500	C31—H31	0.9500
C14—C15	1.36 (2)	C32—H32	0.9500
C14—H14	0.9500	N1—N2	1.284 (14)
C15—C16	1.33 (3)	Cu1—Br4	3.134 (2)
C15—H15	0.9500	Cu1—N1	1.947 (12)
C16—H16	0.9500	N3—N4	1.315 (14)
C17—C18	1.389 (19)	Cu1—N3	1.970 (11)
C17—C22	1.411 (19)	Cu1—O1	1.892 (9)
C17—N3	1.437 (16)	Cu1—O2	1.888 (8)
C18—C19	1.39 (2)		
			
C2—C1—C6	119.5 (13)	C18—C19—H19	120.8
C2—C1—N1	121.2 (12)	C19—C20—C21	124.2 (13)
C6—C1—N1	119.3 (12)	C19—C20—Br5	120.0 (11)
C1—C2—C3	121.0 (13)	C21—C20—Br5	115.6 (11)
C1—C2—Br1	119.8 (11)	C22—C21—C20	116.6 (14)
C3—C2—Br1	119.2 (10)	C22—C21—H21	121.7
C4—C3—C2	117.6 (12)	C20—C21—H21	121.7
C4—C3—H3	121.2	C21—C22—C17	122.8 (12)
C2—C3—H3	121.2	C21—C22—Br6	117.0 (11)
C5—C4—C3	124.9 (13)	C17—C22—Br6	120.1 (10)
C5—C4—Br2	115.3 (11)	N4—C23—C24	123.3 (7)
C3—C4—Br2	119.8 (10)	N4—C23—C28	115.8 (7)
C4—C5—C6	118.3 (13)	C24—C23—C28	120.0
C4—C5—H5	120.8	O2—C24—C25	114.8 (7)
C6—C5—H5	120.8	O2—C24—C23	125.2 (7)
C1—C6—C5	118.7 (12)	C25—C24—C23	120.0
C1—C6—Br3	121.9 (11)	C26—C25—C24	120.0
C5—C6—Br3	119.3 (11)	C26—C25—H25	120.0
N2—C7—C8	126.2 (12)	C24—C25—H25	120.0
N2—C7—C12	114.1 (12)	C25—C26—C27	120.0
C8—C7—C12	119.7 (13)	C25—C26—H26	120.0
O1—C8—C7	125.7 (12)	C27—C26—H26	120.0
O1—C8—C9	115.4 (12)	C28—C27—C26	120.0
C7—C8—C9	118.8 (13)	C28—C27—C32	120.5 (8)
C10—C9—C8	120.9 (15)	C26—C27—C32	119.5 (8)
C10—C9—H9	119.6	C27—C28—C23	120.0
C8—C9—H9	119.6	C27—C28—C29	117.6 (7)
C9—C10—C11	121.1 (15)	C23—C28—C29	122.4 (7)
C9—C10—H10	119.5	C30—C29—C28	122.7 (13)
C11—C10—H10	119.5	C30—C29—H29	118.6
C12—C11—C10	119.5 (15)	C28—C29—H29	118.6
C12—C11—C16	118.0 (17)	C29—C30—C31	118.9 (15)
C10—C11—C16	122.4 (16)	C29—C30—H30	120.6
C11—C12—C7	120.0 (15)	C31—C30—H30	120.6
C11—C12—C13	117.2 (14)	C32—C31—C30	119.9 (14)
C7—C12—C13	122.8 (14)	C32—C31—H31	120.0
C14—C13—C12	121.3 (16)	C30—C31—H31	120.0
C14—C13—H13	119.3	C31—C32—C27	120.2 (12)
C12—C13—H13	119.3	C31—C32—H32	119.9
C15—C14—C13	121.9 (18)	C27—C32—H32	119.9
C15—C14—H14	119.0	N2—N1—C1	111.9 (11)
C13—C14—H14	119.0	N2—N1—Cu1	129.9 (9)
C16—C15—C14	120.1 (17)	C1—N1—Cu1	117.7 (8)
C16—C15—H15	120.0	N1—N2—C7	120.3 (12)
C14—C15—H15	120.0	N4—N3—C17	111.9 (10)
C15—C16—C11	121.3 (18)	N4—N3—Cu1	128.3 (8)
C15—C16—H16	119.4	C17—N3—Cu1	119.4 (8)
C11—C16—H16	119.4	N3—N4—C23	119.1 (10)
C18—C17—C22	115.5 (12)	C8—O1—Cu1	125.8 (9)
C18—C17—N3	119.4 (13)	C24—O2—Cu1	121.9 (7)
C22—C17—N3	125.1 (12)	O2—Cu1—O1	169.4 (4)
C17—C18—C19	122.5 (14)	O2—Cu1—N1	91.3 (4)
C17—C18—Br4	119.0 (11)	O1—Cu1—N1	90.9 (4)
C19—C18—Br4	118.5 (11)	O2—Cu1—N3	87.6 (4)
C20—C19—C18	118.4 (14)	O1—Cu1—N3	92.1 (4)
C20—C19—H19	120.8	N1—Cu1—N3	169.3 (5)
			
C6—C1—C2—C3	0.6 (19)	N3—C17—C22—C21	−178.2 (12)
N1—C1—C2—C3	−177.6 (12)	C18—C17—C22—Br6	174.4 (10)
C6—C1—C2—Br1	179.6 (10)	N3—C17—C22—Br6	−2.0 (18)
N1—C1—C2—Br1	1.3 (16)	N4—C23—C24—O2	−11.8 (10)
C1—C2—C3—C4	0.6 (19)	C28—C23—C24—O2	179.9 (10)
Br1—C2—C3—C4	−178.4 (10)	N4—C23—C24—C25	168.3 (9)
C2—C3—C4—C5	−1 (2)	C28—C23—C24—C25	0.0
C2—C3—C4—Br2	−179.4 (9)	O2—C24—C25—C26	−179.9 (9)
C3—C4—C5—C6	0 (2)	C23—C24—C25—C26	0.0
Br2—C4—C5—C6	178.5 (10)	C24—C25—C26—C27	0.0
C2—C1—C6—C5	−1.6 (19)	C25—C26—C27—C28	0.0
N1—C1—C6—C5	176.7 (12)	C25—C26—C27—C32	179.7 (10)
C2—C1—C6—Br3	177.2 (10)	C26—C27—C28—C23	0.0
N1—C1—C6—Br3	−4.5 (17)	C32—C27—C28—C23	−179.7 (10)
C4—C5—C6—C1	1 (2)	C26—C27—C28—C29	178.7 (10)
C4—C5—C6—Br3	−177.5 (11)	C32—C27—C28—C29	−1.0 (12)
N2—C7—C8—O1	6 (2)	N4—C23—C28—C27	−169.2 (9)
C12—C7—C8—O1	−175.9 (13)	C24—C23—C28—C27	0.0
N2—C7—C8—C9	−175.9 (13)	N4—C23—C28—C29	12.2 (11)
C12—C7—C8—C9	2.2 (19)	C24—C23—C28—C29	−178.6 (11)
O1—C8—C9—C10	176.4 (14)	C27—C28—C29—C30	−2.0 (18)
C7—C8—C9—C10	−2 (2)	C23—C28—C29—C30	176.6 (11)
C8—C9—C10—C11	1 (2)	C28—C29—C30—C31	3 (2)
C9—C10—C11—C12	0 (2)	C29—C30—C31—C32	−1 (2)
C9—C10—C11—C16	−177.0 (15)	C30—C31—C32—C27	−2 (2)
C10—C11—C12—C7	0 (2)	C28—C27—C32—C31	2.9 (17)
C16—C11—C12—C7	177.5 (14)	C26—C27—C32—C31	−176.7 (11)
C10—C11—C12—C13	179.0 (14)	C2—C1—N1—N2	−107.0 (14)
C16—C11—C12—C13	−4 (2)	C6—C1—N1—N2	74.8 (15)
N2—C7—C12—C11	176.9 (12)	C2—C1—N1—Cu1	80.7 (14)
C8—C7—C12—C11	−1 (2)	C6—C1—N1—Cu1	−97.6 (12)
N2—C7—C12—C13	−1.8 (19)	C1—N1—N2—C7	−179.3 (11)
C8—C7—C12—C13	179.9 (13)	Cu1—N1—N2—C7	−8.1 (18)
C11—C12—C13—C14	3 (2)	C8—C7—N2—N1	−3 (2)
C7—C12—C13—C14	−178.0 (15)	C12—C7—N2—N1	179.0 (12)
C12—C13—C14—C15	0 (3)	C18—C17—N3—N4	131.8 (12)
C13—C14—C15—C16	−3 (3)	C22—C17—N3—N4	−52.0 (17)
C14—C15—C16—C11	2 (3)	C18—C17—N3—Cu1	−53.8 (15)
C12—C11—C16—C15	1 (3)	C22—C17—N3—Cu1	122.4 (12)
C10—C11—C16—C15	178.3 (17)	C17—N3—N4—C23	−178.2 (10)
C22—C17—C18—C19	1.8 (19)	Cu1—N3—N4—C23	8.0 (16)
N3—C17—C18—C19	178.4 (12)	C24—C23—N4—N3	20.3 (13)
C22—C17—C18—Br4	−179.3 (9)	C28—C23—N4—N3	−170.9 (8)
N3—C17—C18—Br4	−2.7 (17)	C7—C8—O1—Cu1	2 (2)
C17—C18—C19—C20	0 (2)	C9—C8—O1—Cu1	−176.2 (9)
Br4—C18—C19—C20	−178.8 (11)	C25—C24—O2—Cu1	154.8 (5)
C18—C19—C20—C21	−2 (2)	C23—C24—O2—Cu1	−25.1 (11)
C18—C19—C20—Br5	−176.8 (10)	C24—O2—Cu1—O1	−52 (3)
C19—C20—C21—C22	2 (2)	C24—O2—Cu1—N1	−153.4 (9)
Br5—C20—C21—C22	176.9 (10)	C24—O2—Cu1—N3	37.3 (9)
C20—C21—C22—C17	0 (2)	C8—O1—Cu1—O2	−110 (2)
C20—C21—C22—Br6	−176.3 (10)	C8—O1—Cu1—N1	−8.2 (11)
C18—C17—C22—C21	−1.8 (19)	C8—O1—Cu1—N3	161.5 (11)

### (I) Bis{(E)-1-[(2,4,6-tribromophenyl)diazenyl]naphthalen-2-olato}copper(II) Hydrogen-bond geometry (Å, º)

Cg1 is the centroid of the C27–C32 ring.

**Table:**

*D*—H···*A*	*D*—H	H···*A*	*D*···*A*	*D*—H···*A*
C5—H5···Br6^i^	0.95	2.75	3.546 (15)	142
C3—H3···*Cg*1^ii^	0.95	2.99	3.729 (15)	136

Symmetry codes: (i) *x*, *y*, *z*−1; (ii) −*x*+1, *y*+1/2, −*z*.

### (II) Bis{(E)-1-[(2,4,6-tribromophenyl)diazenyl]naphthalen-2-olato}nickel(II) Crystal data

**Table:**

[Ni(C_16_H_8_Br_3_N_2_O)_2_]	*F*(000) = 980
*M**_r_* = 1026.66	*D*_x_ = 2.067 Mg m^−^^3^
Monoclinic, *P*2_1_/*n*	Mo *K*α radiation, λ = 0.71073 Å
*a* = 11.0909 (6) Å	Cell parameters from 7253 reflections
*b* = 12.4571 (6) Å	θ = 1.0–27.5°
*c* = 12.5382 (7) Å	µ = 7.89 mm^−^^1^
β = 107.820 (2)°	*T* = 173 K
*V* = 1649.17 (15) Å^3^	Prism, black
*Z* = 2	0.30 × 0.22 × 0.06 mm

### (II) Bis{(E)-1-[(2,4,6-tribromophenyl)diazenyl]naphthalen-2-olato}nickel(II) Data collection

**Table:**

Nonius KappaCCD diffractometer	3745 independent reflections
Radiation source: sealed tube	2214 reflections with *I* > 2σ(*I*)
Graphite monochromator	*R*_int_ = 0.094
phi and ω scans	θ_max_ = 27.5°, θ_min_ = 2.9°
Absorption correction: multi-scan (MULABS; Spek, 2009)	*h* = −13→14
*T*_min_ = 0.151, *T*_max_ = 0.317	*k* = −13→16
11360 measured reflections	*l* = −16→16

### (II) Bis{(E)-1-[(2,4,6-tribromophenyl)diazenyl]naphthalen-2-olato}nickel(II) Refinement

**Table:**

Refinement on *F*^2^	Primary atom site location: structure-invariant direct methods
Least-squares matrix: full	Secondary atom site location: difference Fourier map
*R*[*F*^2^ > 2σ(*F*^2^)] = 0.043	Hydrogen site location: inferred from neighbouring sites
*wR*(*F*^2^) = 0.096	H-atom parameters constrained
*S* = 0.95	*w* = 1/[σ^2^(*F*_o_^2^) + (0.034*P*)^2^] where *P* = (*F*_o_^2^ + 2*F*_c_^2^)/3
3745 reflections	(Δ/σ)_max_ < 0.001
205 parameters	Δρ_max_ = 0.57 e Å^−^^3^
0 restraints	Δρ_min_ = −0.66 e Å^−^^3^

### (II) Bis{(E)-1-[(2,4,6-tribromophenyl)diazenyl]naphthalen-2-olato}nickel(II) Special details

**Table:**

Geometry. All esds (except the esd in the dihedral angle between two l.s. planes) are estimated using the full covariance matrix. The cell esds are taken into account individually in the estimation of esds in distances, angles and torsion angles; correlations between esds in cell parameters are only used when they are defined by crystal symmetry. An approximate (isotropic) treatment of cell esds is used for estimating esds involving l.s. planes.

### (II) Bis{(E)-1-[(2,4,6-tribromophenyl)diazenyl]naphthalen-2-olato}nickel(II) Fractional atomic coordinates and isotropic or equivalent isotropic displacement parameters (Å^2^)

**Table:**

	*x*	*y*	*z*	*U*_iso_*/*U*_eq_	
C1	0.2389 (4)	0.4906 (3)	0.3565 (3)	0.0232 (10)	
C2	0.2302 (4)	0.5055 (4)	0.2452 (4)	0.0287 (11)	
C3	0.1396 (4)	0.4527 (4)	0.1602 (4)	0.0321 (12)	
H3	0.1357	0.4619	0.0840	0.038*	
C4	0.0551 (4)	0.3862 (3)	0.1896 (4)	0.0295 (12)	
C5	0.0609 (4)	0.3679 (4)	0.3004 (4)	0.0318 (12)	
H5	0.0028	0.3212	0.3192	0.038*	
C6	0.1547 (4)	0.4204 (4)	0.3819 (3)	0.0277 (11)	
C7	0.3408 (4)	0.6940 (3)	0.5623 (4)	0.0283 (11)	
C8	0.4729 (4)	0.6805 (4)	0.6238 (4)	0.0293 (11)	
C9	0.5307 (4)	0.7555 (4)	0.7110 (4)	0.0335 (12)	
H9	0.6165	0.7462	0.7549	0.040*	
C10	0.4637 (4)	0.8392 (4)	0.7310 (4)	0.0323 (12)	
H10	0.5056	0.8894	0.7871	0.039*	
C11	0.3320 (4)	0.8562 (4)	0.6717 (4)	0.0296 (11)	
C12	0.2707 (4)	0.7808 (4)	0.5879 (3)	0.0272 (11)	
C13	0.1395 (4)	0.7939 (4)	0.5345 (4)	0.0308 (12)	
H13	0.0955	0.7435	0.4795	0.037*	
C14	0.0748 (5)	0.8782 (4)	0.5608 (4)	0.0386 (13)	
H14	−0.0135	0.8852	0.5240	0.046*	
C15	0.1371 (5)	0.9547 (4)	0.6414 (4)	0.0416 (13)	
H15	0.0916	1.0131	0.6591	0.050*	
C16	0.2644 (5)	0.9436 (4)	0.6940 (4)	0.0342 (12)	
H16	0.3075	0.9964	0.7466	0.041*	
N1	0.3320 (3)	0.5483 (3)	0.4453 (3)	0.0251 (9)	
N2	0.2773 (3)	0.6275 (3)	0.4773 (3)	0.0278 (9)	
O1	0.5435 (3)	0.6061 (2)	0.6047 (2)	0.0328 (8)	
Ni	0.5000	0.5000	0.5000	0.0271 (2)	
Br1	0.16663 (5)	0.39355 (4)	0.53300 (4)	0.04164 (17)	
Br2	0.34269 (5)	0.60009 (4)	0.20681 (4)	0.04697 (18)	
Br3	−0.07417 (5)	0.31689 (4)	0.07466 (4)	0.05057 (19)	

### (II) Bis{(E)-1-[(2,4,6-tribromophenyl)diazenyl]naphthalen-2-olato}nickel(II) Atomic displacement parameters (Å^2^)

**Table:**

	*U*^11^	*U*^22^	*U*^33^	*U*^12^	*U*^13^	*U*^23^
C1	0.014 (2)	0.027 (2)	0.024 (2)	0.003 (2)	−0.0014 (19)	−0.004 (2)
C2	0.023 (2)	0.031 (3)	0.027 (2)	−0.007 (2)	0.000 (2)	−0.002 (2)
C3	0.029 (3)	0.037 (3)	0.025 (2)	0.004 (2)	0.001 (2)	0.000 (2)
C4	0.020 (2)	0.027 (3)	0.033 (3)	0.002 (2)	−0.005 (2)	−0.008 (2)
C5	0.028 (3)	0.030 (3)	0.034 (3)	−0.002 (2)	0.005 (2)	0.002 (2)
C6	0.025 (3)	0.031 (3)	0.022 (2)	0.006 (2)	0.000 (2)	0.001 (2)
C7	0.024 (3)	0.030 (3)	0.025 (2)	0.001 (2)	−0.002 (2)	−0.004 (2)
C8	0.025 (3)	0.034 (3)	0.025 (2)	−0.006 (2)	0.002 (2)	−0.004 (2)
C9	0.023 (3)	0.037 (3)	0.033 (3)	−0.003 (2)	−0.003 (2)	−0.009 (2)
C10	0.032 (3)	0.034 (3)	0.027 (2)	−0.009 (2)	0.002 (2)	−0.002 (2)
C11	0.033 (3)	0.028 (3)	0.026 (2)	−0.002 (2)	0.006 (2)	−0.001 (2)
C12	0.024 (3)	0.032 (3)	0.024 (2)	0.003 (2)	0.004 (2)	−0.002 (2)
C13	0.029 (3)	0.029 (3)	0.031 (2)	0.006 (2)	0.004 (2)	−0.003 (2)
C14	0.025 (3)	0.050 (3)	0.035 (3)	0.009 (3)	0.001 (2)	0.004 (3)
C15	0.043 (3)	0.040 (3)	0.042 (3)	0.017 (3)	0.011 (3)	0.001 (3)
C16	0.042 (3)	0.031 (3)	0.030 (3)	0.000 (2)	0.011 (2)	−0.003 (2)
N1	0.018 (2)	0.029 (2)	0.0214 (19)	−0.0025 (17)	−0.0034 (16)	−0.0038 (17)
N2	0.026 (2)	0.025 (2)	0.026 (2)	0.0012 (18)	−0.0019 (18)	−0.0021 (17)
O1	0.0247 (18)	0.0333 (19)	0.0316 (18)	0.0022 (15)	−0.0044 (15)	−0.0132 (14)
Ni	0.0197 (4)	0.0297 (5)	0.0255 (4)	0.0004 (4)	−0.0026 (4)	−0.0040 (4)
Br1	0.0424 (3)	0.0495 (4)	0.0289 (3)	−0.0039 (3)	0.0049 (2)	0.0067 (2)
Br2	0.0386 (3)	0.0609 (4)	0.0378 (3)	−0.0170 (3)	0.0063 (3)	0.0049 (3)
Br3	0.0419 (3)	0.0534 (4)	0.0427 (3)	−0.0125 (3)	−0.0073 (3)	−0.0172 (3)

### (II) Bis{(E)-1-[(2,4,6-tribromophenyl)diazenyl]naphthalen-2-olato}nickel(II) Geometric parameters (Å, º)

**Table:**

C1—C2	1.382 (6)	C9—H9	0.9500
C1—C6	1.385 (6)	C10—C11	1.437 (6)
C1—N1	1.455 (5)	C10—H10	0.9500
C2—C3	1.387 (6)	C11—C16	1.399 (7)
C2—Br2	1.883 (5)	C11—C12	1.419 (6)
C3—C4	1.382 (7)	C12—C13	1.413 (6)
C3—H3	0.9500	C13—C14	1.367 (6)
C4—C5	1.389 (6)	C13—H13	0.9500
C4—Br3	1.901 (4)	C14—C15	1.407 (7)
C5—C6	1.381 (6)	C14—H14	0.9500
C5—H5	0.9500	C15—C16	1.370 (7)
C6—Br1	1.888 (4)	C15—H15	0.9500
C7—N2	1.363 (5)	C16—H16	0.9500
C7—C12	1.425 (6)	N1—N2	1.285 (5)
C7—C8	1.441 (6)	Ni—N1	1.876 (3)
C8—O1	1.281 (5)	Ni—O1	1.821 (3)
C8—C9	1.431 (6)	Ni—O1^i^	1.821 (3)
C9—C10	1.348 (6)	Ni—N1^i^	1.877 (3)
			
C2—C1—C6	118.3 (4)	C16—C11—C12	119.9 (4)
C2—C1—N1	121.3 (4)	C16—C11—C10	122.2 (4)
C6—C1—N1	120.4 (4)	C12—C11—C10	117.8 (4)
C1—C2—C3	121.5 (4)	C13—C12—C11	117.7 (4)
C1—C2—Br2	119.7 (3)	C13—C12—C7	122.4 (4)
C3—C2—Br2	118.8 (4)	C11—C12—C7	119.9 (4)
C4—C3—C2	118.0 (4)	C14—C13—C12	121.0 (4)
C4—C3—H3	121.0	C14—C13—H13	119.5
C2—C3—H3	121.0	C12—C13—H13	119.5
C3—C4—C5	122.5 (4)	C13—C14—C15	121.0 (4)
C3—C4—Br3	119.0 (4)	C13—C14—H14	119.5
C5—C4—Br3	118.5 (4)	C15—C14—H14	119.5
C6—C5—C4	117.1 (4)	C16—C15—C14	119.0 (5)
C6—C5—H5	121.4	C16—C15—H15	120.5
C4—C5—H5	121.4	C14—C15—H15	120.5
C5—C6—C1	122.5 (4)	C15—C16—C11	121.3 (4)
C5—C6—Br1	117.7 (4)	C15—C16—H16	119.4
C1—C6—Br1	119.8 (3)	C11—C16—H16	119.4
N2—C7—C12	116.7 (4)	N2—N1—C1	109.0 (3)
N2—C7—C8	123.0 (4)	N2—N1—Ni	130.0 (3)
C12—C7—C8	120.2 (4)	C1—N1—Ni	120.9 (3)
O1—C8—C9	117.3 (4)	N1—N2—C7	122.0 (4)
O1—C8—C7	124.2 (4)	C8—O1—Ni	128.1 (3)
C9—C8—C7	118.5 (4)	O1—Ni—O1^i^	180
C10—C9—C8	120.3 (4)	O1—Ni—N1	92.59 (14)
C10—C9—H9	119.9	O1^i^—Ni—N1	87.41 (14)
C8—C9—H9	119.9	O1—Ni—N1^i^	87.41 (14)
C9—C10—C11	123.2 (4)	O1^i^—Ni—N1^i^	92.59 (14)
C9—C10—H10	118.4	N1—Ni—N1^i^	180
C11—C10—H10	118.4		
			
C6—C1—C2—C3	0.3 (7)	C10—C11—C12—C7	−2.3 (7)
N1—C1—C2—C3	−178.4 (4)	N2—C7—C12—C13	4.6 (7)
C6—C1—C2—Br2	−180.0 (3)	C8—C7—C12—C13	−176.3 (4)
N1—C1—C2—Br2	1.3 (6)	N2—C7—C12—C11	−177.0 (4)
C1—C2—C3—C4	1.7 (7)	C8—C7—C12—C11	2.1 (7)
Br2—C2—C3—C4	−178.0 (3)	C11—C12—C13—C14	1.7 (7)
C2—C3—C4—C5	−2.3 (7)	C7—C12—C13—C14	−179.9 (5)
C2—C3—C4—Br3	177.6 (3)	C12—C13—C14—C15	0.3 (8)
C3—C4—C5—C6	0.8 (7)	C13—C14—C15—C16	−0.1 (8)
Br3—C4—C5—C6	−179.1 (3)	C14—C15—C16—C11	−2.2 (8)
C4—C5—C6—C1	1.4 (7)	C12—C11—C16—C15	4.2 (7)
C4—C5—C6—Br1	−178.4 (3)	C10—C11—C16—C15	−175.9 (4)
C2—C1—C6—C5	−1.9 (7)	C2—C1—N1—N2	100.7 (5)
N1—C1—C6—C5	176.8 (4)	C6—C1—N1—N2	−78.1 (5)
C2—C1—C6—Br1	177.8 (3)	C2—C1—N1—Ni	−82.2 (5)
N1—C1—C6—Br1	−3.4 (5)	C6—C1—N1—Ni	99.1 (4)
N2—C7—C8—O1	0.5 (7)	C1—N1—N2—C7	178.0 (4)
C12—C7—C8—O1	−178.6 (4)	Ni—N1—N2—C7	1.1 (6)
N2—C7—C8—C9	179.6 (4)	C12—C7—N2—N1	178.2 (4)
C12—C7—C8—C9	0.5 (7)	C8—C7—N2—N1	−0.9 (7)
O1—C8—C9—C10	176.2 (5)	C9—C8—O1—Ni	−179.4 (3)
C7—C8—C9—C10	−2.9 (7)	C7—C8—O1—Ni	−0.3 (7)
C8—C9—C10—C11	2.8 (7)	C8—O1—Ni—N1	0.4 (4)
C9—C10—C11—C16	179.9 (5)	C8—O1—Ni—N1^i^	−179.6 (4)
C9—C10—C11—C12	−0.2 (7)	N2—N1—Ni—O1	−0.8 (4)
C16—C11—C12—C13	−3.9 (7)	C1—N1—Ni—O1	−177.3 (3)
C10—C11—C12—C13	176.2 (4)	N2—N1—Ni—O1^i^	179.2 (4)
C16—C11—C12—C7	177.6 (4)	C1—N1—Ni—O1^i^	2.7 (3)

Symmetry code: (i) −*x*+1, −*y*+1, −*z*+1.

### (II) Bis{(E)-1-[(2,4,6-tribromophenyl)diazenyl]naphthalen-2-olato}nickel(II) Hydrogen-bond geometry (Å, º)

Cg2 is the centroid of the C1–C6 ring.

**Table:**

*D*—H···*A*	*D*—H	H···*A*	*D*···*A*	*D*—H···*A*
C10—H10···*Cg*2^ii^	0.95	2.71	3.391 (5)	130

Symmetry code: (ii) *x*+1/2, −*y*+3/2, *z*+1/2.

### (III) Bis{(E)-1-[(2,4,6-tribromophenyl)diazenyl]naphthalen-2-olato}palladium(II) Crystal data

**Table:**

[Pd(C_16_H_8_Br_3_N_2_O)_2_]	*F*(000) = 1016
*M**_r_* = 1074.35	*D*_x_ = 2.142 Mg m^−^^3^
Monoclinic, *P*2_1_/*n*	Cu *K*α radiation, λ = 1.54186 Å
*a* = 11.1896 (8) Å	Cell parameters from 3651 reflections
*b* = 12.4540 (8) Å	θ = 2.1–22.3°
*c* = 12.5511 (9) Å	µ = 13.23 mm^−^^1^
β = 107.749 (5)°	*T* = 200 K
*V* = 1665.8 (2) Å^3^	Square plate, dark red
*Z* = 2	0.12 × 0.09 × 0.03 mm

### (III) Bis{(E)-1-[(2,4,6-tribromophenyl)diazenyl]naphthalen-2-olato}palladium(II) Data collection

**Table:**

STOE IPDS 2T diffractometer	2895 independent reflections
Radiation source: Genix-Cu,3D	2371 reflections with *I* > 2σ(*I*)
Graphite monochromator	*R*_int_ = 0.142
Detector resolution: 6.67 pixels mm^-1^	θ_max_ = 67.7°, θ_min_ = 5.5°
rotation method scans	*h* = −13→12
Absorption correction: multi-scan (MULABS; Spek, 2009)	*k* = −14→14
*T*_min_ = 0.360, *T*_max_ = 1.000	*l* = −14→15
13003 measured reflections	

### (III) Bis{(E)-1-[(2,4,6-tribromophenyl)diazenyl]naphthalen-2-olato}palladium(II) Refinement

**Table:**

Refinement on *F*^2^	Secondary atom site location: difference Fourier map
Least-squares matrix: full	Hydrogen site location: inferred from neighbouring sites
*R*[*F*^2^ > 2σ(*F*^2^)] = 0.057	H-atom parameters constrained
*wR*(*F*^2^) = 0.170	*w* = 1/[σ^2^(*F*_o_^2^) + (0.1019*P*)^2^ + 0.8121*P*] where *P* = (*F*_o_^2^ + 2*F*_c_^2^)/3
*S* = 1.11	(Δ/σ)_max_ < 0.001
2895 reflections	Δρ_max_ = 0.88 e Å^−^^3^
206 parameters	Δρ_min_ = −1.10 e Å^−^^3^
0 restraints	Extinction correction: SHELXL, Fc^*^=kFc[1+0.001xFc^2^λ^3^/sin(2θ)]^-1/4^
Primary atom site location: structure-invariant direct methods	Extinction coefficient: 0.0040 (4)

### (III) Bis{(E)-1-[(2,4,6-tribromophenyl)diazenyl]naphthalen-2-olato}palladium(II) Special details

**Table:**

Geometry. All esds (except the esd in the dihedral angle between two l.s. planes) are estimated using the full covariance matrix. The cell esds are taken into account individually in the estimation of esds in distances, angles and torsion angles; correlations between esds in cell parameters are only used when they are defined by crystal symmetry. An approximate (isotropic) treatment of cell esds is used for estimating esds involving l.s. planes.

### (III) Bis{(E)-1-[(2,4,6-tribromophenyl)diazenyl]naphthalen-2-olato}palladium(II) Fractional atomic coordinates and isotropic or equivalent isotropic displacement parameters (Å^2^)

**Table:**

	*x*	*y*	*z*	*U*_iso_*/*U*_eq_	
Br1	0.16311 (8)	0.39498 (7)	0.53262 (6)	0.0659 (3)	
Br2	0.33399 (8)	0.60480 (8)	0.20631 (7)	0.0737 (4)	
Br3	−0.07036 (8)	0.31295 (7)	0.07577 (7)	0.0754 (4)	
Pd1	0.5000	0.5000	0.5000	0.0456 (3)	
O1	0.5428 (4)	0.6130 (4)	0.6155 (4)	0.0573 (12)	
N1	0.3223 (5)	0.5521 (4)	0.4432 (4)	0.0468 (12)	
N2	0.2730 (5)	0.6310 (4)	0.4798 (4)	0.0486 (12)	
C1	0.2323 (6)	0.4941 (5)	0.3582 (5)	0.0471 (15)	
C2	0.2246 (6)	0.5074 (5)	0.2447 (5)	0.0482 (15)	
C3	0.1354 (7)	0.4532 (6)	0.1598 (5)	0.0563 (17)	
H3	0.1311	0.4629	0.0836	0.068*	
C4	0.0544 (7)	0.3855 (5)	0.1889 (6)	0.0532 (16)	
C5	0.0597 (6)	0.3675 (5)	0.2996 (6)	0.0495 (15)	
H5	0.0032	0.3195	0.3184	0.059*	
C6	0.1513 (6)	0.4227 (5)	0.3817 (5)	0.0474 (14)	
C7	0.3371 (6)	0.6961 (5)	0.5647 (5)	0.0464 (14)	
C8	0.4672 (6)	0.6842 (6)	0.6279 (5)	0.0523 (15)	
C9	0.5192 (7)	0.7634 (6)	0.7146 (6)	0.0570 (17)	
H9	0.6039	0.7562	0.7601	0.068*	
C10	0.4511 (7)	0.8468 (6)	0.7323 (6)	0.0604 (18)	
H10	0.4904	0.8983	0.7877	0.072*	
C11	0.3214 (7)	0.8605 (6)	0.6705 (6)	0.0536 (16)	
C12	0.2640 (7)	0.7831 (5)	0.5895 (5)	0.0505 (15)	
C13	0.1345 (7)	0.7932 (6)	0.5352 (6)	0.0583 (17)	
H13	0.0933	0.7427	0.4793	0.070*	
C14	0.0678 (8)	0.8748 (7)	0.5619 (7)	0.070 (2)	
H14	−0.0202	0.8783	0.5263	0.084*	
C15	0.1257 (8)	0.9538 (7)	0.6406 (6)	0.071 (2)	
H15	0.0780	1.0109	0.6573	0.085*	
C16	0.2509 (8)	0.9469 (7)	0.6922 (7)	0.0652 (19)	
H16	0.2917	1.0010	0.7438	0.078*	

### (III) Bis{(E)-1-[(2,4,6-tribromophenyl)diazenyl]naphthalen-2-olato}palladium(II) Atomic displacement parameters (Å^2^)

**Table:**

	*U*^11^	*U*^22^	*U*^33^	*U*^12^	*U*^13^	*U*^23^
Br1	0.0649 (6)	0.0817 (6)	0.0436 (5)	−0.0081 (4)	0.0055 (4)	0.0092 (3)
Br2	0.0614 (6)	0.0997 (7)	0.0531 (5)	−0.0261 (4)	0.0070 (4)	0.0073 (4)
Br3	0.0648 (6)	0.0834 (6)	0.0603 (6)	−0.0172 (4)	−0.0073 (4)	−0.0226 (4)
Pd1	0.0353 (4)	0.0550 (4)	0.0369 (4)	−0.0014 (3)	−0.0032 (3)	−0.0038 (3)
O1	0.042 (3)	0.065 (3)	0.052 (3)	0.003 (2)	−0.005 (2)	−0.009 (2)
N1	0.042 (3)	0.053 (3)	0.037 (3)	−0.007 (2)	−0.001 (2)	−0.004 (2)
N2	0.040 (3)	0.054 (3)	0.042 (3)	−0.004 (2)	−0.003 (2)	0.000 (2)
C1	0.038 (3)	0.054 (3)	0.037 (3)	0.007 (3)	−0.007 (3)	0.001 (3)
C2	0.036 (3)	0.061 (4)	0.037 (3)	0.000 (3)	−0.005 (3)	0.002 (3)
C3	0.049 (4)	0.073 (4)	0.038 (3)	0.004 (3)	−0.001 (3)	−0.001 (3)
C4	0.045 (4)	0.056 (4)	0.048 (4)	−0.005 (3)	−0.002 (3)	−0.005 (3)
C5	0.043 (4)	0.047 (3)	0.054 (4)	−0.002 (3)	0.008 (3)	0.002 (3)
C6	0.042 (3)	0.049 (3)	0.040 (3)	−0.002 (3)	−0.003 (3)	0.000 (3)
C7	0.035 (3)	0.057 (3)	0.042 (3)	−0.001 (3)	0.004 (3)	−0.005 (3)
C8	0.045 (4)	0.063 (4)	0.040 (3)	−0.007 (3)	0.001 (3)	0.001 (3)
C9	0.046 (4)	0.070 (4)	0.047 (4)	−0.004 (3)	0.002 (3)	−0.012 (3)
C10	0.059 (4)	0.070 (4)	0.043 (4)	−0.014 (4)	0.002 (3)	−0.010 (3)
C11	0.053 (4)	0.060 (4)	0.046 (4)	0.001 (3)	0.012 (3)	−0.002 (3)
C12	0.048 (4)	0.057 (4)	0.043 (3)	−0.007 (3)	0.008 (3)	−0.001 (3)
C13	0.046 (4)	0.070 (4)	0.052 (4)	0.000 (3)	0.004 (3)	−0.010 (3)
C14	0.056 (5)	0.080 (5)	0.064 (5)	0.002 (4)	0.004 (4)	−0.003 (4)
C15	0.074 (6)	0.072 (5)	0.060 (5)	0.019 (4)	0.009 (4)	0.001 (4)
C16	0.069 (5)	0.065 (4)	0.057 (4)	−0.002 (4)	0.013 (4)	0.002 (4)

### (III) Bis{(E)-1-[(2,4,6-tribromophenyl)diazenyl]naphthalen-2-olato}palladium(II) Geometric parameters (Å, º)

**Table:**

Br1—C6	1.889 (6)	C5—H5	0.9500
Br2—C2	1.887 (7)	C7—C8	1.437 (9)
Br3—C4	1.890 (7)	C7—C12	1.448 (9)
Pd1—O1	1.972 (5)	C8—C9	1.452 (10)
Pd1—O1^i^	1.972 (5)	C9—C10	1.346 (10)
Pd1—N1^i^	2.004 (5)	C9—H9	0.9500
Pd1—N1	2.004 (5)	C10—C11	1.432 (10)
O1—C8	1.268 (8)	C10—H10	0.9500
N1—N2	1.279 (8)	C11—C12	1.406 (10)
N1—C1	1.422 (8)	C11—C16	1.409 (11)
N2—C7	1.356 (8)	C12—C13	1.406 (10)
C1—C6	1.365 (9)	C13—C14	1.362 (11)
C1—C2	1.410 (9)	C13—H13	0.9500
C2—C3	1.393 (9)	C14—C15	1.405 (12)
C3—C4	1.367 (10)	C14—H14	0.9500
C3—H3	0.9500	C15—C16	1.355 (11)
C4—C5	1.390 (10)	C15—H15	0.9500
C5—C6	1.394 (9)	C16—H16	0.9500
			
O1—Pd1—O1^i^	180.0	N2—C7—C12	114.7 (6)
O1—Pd1—N1^i^	88.7 (2)	C8—C7—C12	120.1 (6)
O1^i^—Pd1—N1^i^	91.3 (2)	O1—C8—C7	127.3 (6)
O1—Pd1—N1	91.3 (2)	O1—C8—C9	115.9 (6)
O1^i^—Pd1—N1	88.7 (2)	C7—C8—C9	116.8 (6)
N1^i^—Pd1—N1	180.0	C10—C9—C8	122.0 (7)
C8—O1—Pd1	124.6 (4)	C10—C9—H9	119.0
N2—N1—C1	112.0 (5)	C8—C9—H9	119.0
N2—N1—Pd1	127.7 (4)	C9—C10—C11	122.3 (6)
C1—N1—Pd1	120.1 (4)	C9—C10—H10	118.9
N1—N2—C7	123.9 (5)	C11—C10—H10	118.9
C6—C1—C2	117.1 (6)	C12—C11—C16	120.3 (7)
C6—C1—N1	122.3 (6)	C12—C11—C10	118.3 (6)
C2—C1—N1	120.6 (6)	C16—C11—C10	121.4 (7)
C3—C2—C1	121.7 (6)	C13—C12—C11	117.7 (6)
C3—C2—Br2	119.0 (5)	C13—C12—C7	121.9 (6)
C1—C2—Br2	119.3 (5)	C11—C12—C7	120.4 (6)
C4—C3—C2	118.2 (6)	C14—C13—C12	120.6 (7)
C4—C3—H3	120.9	C14—C13—H13	119.7
C2—C3—H3	120.9	C12—C13—H13	119.7
C3—C4—C5	122.5 (6)	C13—C14—C15	121.7 (8)
C3—C4—Br3	119.5 (5)	C13—C14—H14	119.1
C5—C4—Br3	118.0 (5)	C15—C14—H14	119.1
C4—C5—C6	117.2 (6)	C16—C15—C14	118.7 (8)
C4—C5—H5	121.4	C16—C15—H15	120.7
C6—C5—H5	121.4	C14—C15—H15	120.7
C1—C6—C5	123.3 (6)	C15—C16—C11	120.9 (7)
C1—C6—Br1	119.2 (5)	C15—C16—H16	119.5
C5—C6—Br1	117.5 (5)	C11—C16—H16	119.5
N2—C7—C8	125.2 (6)		
			
C1—N1—N2—C7	176.7 (6)	Pd1—O1—C8—C9	−177.0 (5)
Pd1—N1—N2—C7	1.8 (9)	N2—C7—C8—O1	0.2 (11)
N2—N1—C1—C6	−77.8 (7)	C12—C7—C8—O1	−179.5 (6)
Pd1—N1—C1—C6	97.6 (6)	N2—C7—C8—C9	179.0 (6)
N2—N1—C1—C2	103.1 (7)	C12—C7—C8—C9	−0.7 (9)
Pd1—N1—C1—C2	−81.6 (6)	O1—C8—C9—C10	176.2 (7)
C6—C1—C2—C3	2.2 (9)	C7—C8—C9—C10	−2.7 (10)
N1—C1—C2—C3	−178.6 (6)	C8—C9—C10—C11	2.8 (11)
C6—C1—C2—Br2	−180.0 (5)	C9—C10—C11—C12	0.7 (11)
N1—C1—C2—Br2	−0.8 (8)	C9—C10—C11—C16	178.4 (7)
C1—C2—C3—C4	−0.1 (10)	C16—C11—C12—C13	−2.3 (10)
Br2—C2—C3—C4	−178.0 (5)	C10—C11—C12—C13	175.4 (7)
C2—C3—C4—C5	−1.4 (11)	C16—C11—C12—C7	178.2 (6)
C2—C3—C4—Br3	178.6 (5)	C10—C11—C12—C7	−4.1 (10)
C3—C4—C5—C6	0.8 (10)	N2—C7—C12—C13	4.8 (9)
Br3—C4—C5—C6	−179.2 (5)	C8—C7—C12—C13	−175.4 (6)
C2—C1—C6—C5	−2.9 (10)	N2—C7—C12—C11	−175.7 (6)
N1—C1—C6—C5	177.9 (6)	C8—C7—C12—C11	4.1 (9)
C2—C1—C6—Br1	176.4 (5)	C11—C12—C13—C14	−0.8 (11)
N1—C1—C6—Br1	−2.8 (8)	C7—C12—C13—C14	178.7 (7)
C4—C5—C6—C1	1.5 (10)	C12—C13—C14—C15	2.6 (13)
C4—C5—C6—Br1	−177.8 (5)	C13—C14—C15—C16	−1.2 (13)
N1—N2—C7—C8	−2.2 (10)	C14—C15—C16—C11	−2.0 (12)
N1—N2—C7—C12	177.6 (6)	C12—C11—C16—C15	3.8 (11)
Pd1—O1—C8—C7	1.8 (10)	C10—C11—C16—C15	−173.9 (7)

Symmetry code: (i) −*x*+1, −*y*+1, −*z*+1.

### (III) Bis{(E)-1-[(2,4,6-tribromophenyl)diazenyl]naphthalen-2-olato}palladium(II) Hydrogen-bond geometry (Å, º)

Cg2 is the centroid of ring C1-C6.

**Table:**

*D*—H···*A*	*D*—H	H···*A*	*D*···*A*	*D*—H···*A*
C10—H10···*Cg*2^ii^	0.95	2.70	3.371 (8)	128

Symmetry code: (ii) *x*+1/2, −*y*+3/2, *z*+1/2.
